# From Thermal Springs to Saline Solutions: A Scoping Review of Salt-Based Oral Healthcare Interventions

**DOI:** 10.3390/dj14010032

**Published:** 2026-01-05

**Authors:** Elisabetta Ferrara, Manela Scaramuzzino, Biagio Rapone, Giovanna Murmura, Bruna Sinjari

**Affiliations:** 1 Department of Human Sciences, Law, and Economics, Telematic University “Leonardo Da Vinci” (UNIDAV), Piazza San Rocco, 2, Torrevecchia Teatina, 66100 Chieti, Italy; 2 Thermal Medical Center of Saturnia, 58014 Grosseto, Italy; m.scaramuzzino@termedicasciana.it; 3 Interdisciplinary Department of Medicine, Section of Dentistry, “Aldo Moro” University of Bari, 70124 Bari, Italy; biagio.rapone@uniba.it; 4 Department of Innovative Technologies in Medicine and Dentistry, “G. D’Annunzio” University, 67100 Chieti-Pescara, Italy; giovanna.murmura@unich.it (G.M.); bruna.sinjari@unich.it (B.S.)

**Keywords:** thermal water, mineral water, saline solution, sodium chloride, mouthwash, oral health

## Abstract

**Background**: Therapeutic applications of saline solutions in oral healthcare range from mineral waters to standardized sodium chloride preparations. Despite widespread traditional use, their scientific foundation remains inadequately characterized. This scoping review aimed to systematically map the available evidence for salt-based oral health interventions, characterize study populations and outcomes, and identify research gaps to guide future investigations. **Methods**: Following JBI methodology and PRISMA-ScR guidelines, four electronic databases (PubMed, Scopus, Web of Science, and Cochrane Library) were systematically searched for publications from 2000 to 2025. Studies were classified along a spectrum from geological mineral waters to artificial preparations. Narrative synthesis was employed with systematic gap identification. **Results**: Seventeen studies met inclusion criteria, with a median sample size of 41 participants and a median follow-up of 4 weeks. Evidence distribution revealed concentration on hypersaline Dead Sea derivatives (n = 7, 41%) and European thermal waters (n = 5, 29%), with limited representation of marine-derived (n = 1, 6%) and simple saline solutions (n = 3, 18%). Reported outcomes included periodontal parameters, xerostomia symptoms, viral load, mucositis severity, and dentin hypersensitivity, with variable methodological quality across studies. Heterogeneity in interventions, comparators, and outcome measures precluded direct comparisons. **Conclusions**: The current evidence base for salt-based oral interventions remains limited and methodologically heterogeneous. While preliminary findings suggest potential applications across multiple clinical domains, small sample sizes, short follow-up periods, and inconsistent outcome measures preclude definitive recommendations. Standardized protocols and adequately powered trials are needed before evidence-based clinical integration.

## 1. Introduction

Chlorhexidine-based mouthwashes remain the gold standard for chemical plaque control, demonstrating consistent efficacy in reducing bacterial adhesion and gingival inflammation [1,2]. However, accumulating evidence documents clinically relevant adverse effects, including extrinsic dental staining, supragingival calculus formation, taste alteration, and hypersensitivity reactions, which limit long-term patient compliance [3,4]. Furthermore, emerging research indicates that broad-spectrum antimicrobial agents may induce dysbiotic shifts in the oral microbiome, with potential downstream consequences, including impaired enterosalivary nitrate-nitrite-nitric oxide pathway function and associated cardiovascular implications [5,6].

These concerns have stimulated renewed interest in alternative antimicrobial and anti-inflammatory approaches, among which warrant systematic investigation given their extensive ethnopharmacological history and favorable safety profile [7]. The term “saline-based interventions” encompasses a compositionally heterogeneous spectrum ranging from geologically complex mineral waters to standardized sodium chloride preparations. Natural thermal and mineral waters acquire distinctive ionic signatures through prolonged hydrothermal circulation within specific geological formations, yielding solutions characterized by variable concentrations of dissolved minerals, trace elements, and potentially bioactive compounds [8,9]. Hypersaline environments exemplified by the Dead Sea basin, with total dissolved solids exceeding 340 g/L, present extreme ionic compositions—notably elevated magnesium, potassium, and bromide concentrations—hypothesized to exert therapeutic effects through osmotic bacterial stress, enhanced epithelial penetration, and anti-inflammatory mineral activity [10,11]. European thermal medicine centers, particularly in France (Avène, La Roche-Posay), Italy (Salsomaggiore, Saturnia), and Germany (Baden-Baden), have maintained structured therapeutic protocols for centuries, with documented efficacy in dermatological and rheumatological applications [12,13].

Nevertheless, systematic investigation of oral health applications remains notably limited. Marine-derived preparations occupy an intermediate position along this compositional spectrum, offering the reproducibility of standard seawater salinity (approximately 35 g/L total dissolved solids) with naturally occurring trace element profiles less variable than those of geologically constrained thermal sources [14,15]. Simple saline solutions represent the opposite pole: standardized sodium chloride concentrations eliminate compositional variability while preserving fundamental osmotic mechanisms of bacterial inhibition and wound healing promotion [16]. This latter category holds particular relevance for resource-limited settings where commercial antimicrobial formulations remain economically inaccessible. Despite centuries of empirical application across diverse cultural contexts, the scientific evidence base for salt-based oral interventions remains inadequately characterized. While the balneotherapy literature addressing systemic and dermatological applications is extensive, comparatively few controlled studies have evaluated oral health outcomes specifically.

Prior reviews have typically addressed isolated intervention categories or specific clinical conditions without systematic mapping across the full intervention spectrum. The heterogeneous nature of this literature—encompassing diverse water sources, delivery modalities, clinical populations, and outcome measures—renders conventional systematic review methodology with quantitative synthesis premature.

In this context, the present scoping review aimed to: (1) systematically map the extent and nature of research evidence concerning salt-based interventions for oral health applications; (2) characterize study populations, intervention types, comparators, and outcomes assessed; and (3) identify gaps in the existing literature to inform priorities for future primary research.

## 2. Materials and Methods

### 2.1. Search Strategy

This scoping review was conducted in accordance with the Preferred Reporting Items for Systematic Reviews and Meta-Analyses extension for Scoping Reviews (PRISMA-ScR) guidelines [17] (see Supplementary Materials, Checklist S1). The protocol was prospectively registered on the Open Science Framework (OSF) (DOI: 10.17605/OSF.IO/3XHR6). A systematic search was conducted across four electronic databases: PubMed/MEDLINE, Scopus, Web of Science Core Collection, and Cochrane Library. The initial search was performed in October 2024 and updated on November 2025 to capture recent publications. Searches were limited to publications from January 2000 to December 2025 and to studies published in English, Italian, French, German, Spanish, or Portuguese.

The search strategy was developed in consultation with a medical librarian and combined controlled vocabulary (MeSH terms, Emtree) with free-text terms. No filters for study design were applied to maximize sensitivity. The complete search strategies for all databases are provided in Supplementary Table S1. The PubMed search strategy was as follows:

((“thermal water*”[tiab] OR “mineral water*”[tiab] OR “spa water*”[tiab] OR “balneotherapy”[tiab] OR “Dead Sea”[tiab] OR “saline solution*”[tiab] OR “salt water”[tiab] OR “saltwater”[tiab] OR “sodium chloride solution*”[tiab] OR “hypertonic saline”[tiab] OR “marine water”[tiab] OR “seawater”[tiab] OR “sea water”[tiab]) AND (“oral health”[tiab] OR “oral hygiene”[tiab] OR “periodontal”[tiab] OR “periodontitis”[tiab] OR “gingivitis”[tiab] OR “gingival”[tiab] OR “mouthwash*”[tiab] OR “mouth rinse*”[tiab] OR “mouthrinse*”[tiab] OR “oral rinse*”[tiab] OR “dental”[tiab] OR “oral mucosa”[tiab] OR “stomatitis”[tiab] OR “mucositis”[tiab] OR “plaque”[tiab] OR “caries”[tiab]))

This strategy was adapted for each database according to platform-specific syntax requirements. Grey literature was searched through ClinicalTrials.gov, WHO International Clinical Trials Registry Platform (ICTRP), and OpenGrey. Reference lists of included studies and relevant reviews were hand-searched to identify additional eligible publications. Conference abstracts were excluded due to insufficient methodological detail for quality assessment.

In accordance with the SWiM reporting framework, outcome domains were predefined and included periodontal parameters, xerostomia and salivary function, antimicrobial and antiviral effects, mucositis, dentin hypersensitivity, and whitening and remineralization outcomes.

**Figure 1:**
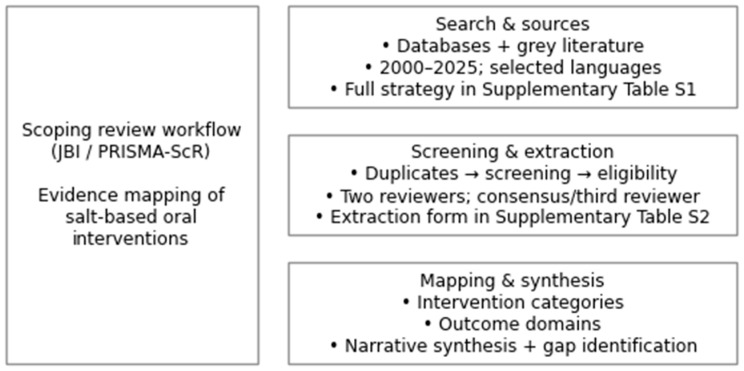
Methodological workflow of the scoping review.

Figure 1 outlines the methodological workflow of the scoping review, from literature search and screening to evidence mapping and synthesis. 

This figure illustrates the methodological workflow of the scoping review conducted in accordance with JBI and PRISMA-ScR guidance, including the search strategy and sources, screening and data extraction process, and evidence mapping and synthesis steps. The complete database-specific strategies are reported in the Supplementary Materials (Table S1).

### 2.2. Eligibility Criteria

Eligibility criteria were defined using the Population, Concept, Context (PCC) framework recommended for scoping reviews.

*Population*: Human participants of any age, including healthy individuals and those with oral or systemic conditions. In vitro and animal studies were excluded unless they included a human clinical component.

*Concept*: Interventions using salt-based or mineral-rich water solutions for oral health purposes. This included hypersaline waters (Dead Sea derivatives), thermal mineral waters, marine-derived solutions, simple saline solutions, and sea salt formulations. Interventions could be administered as mouthwashes, rinses, sprays, irrigations, toothpastes, or gels. Studies examining sodium chloride solely as a vehicle or negative control without therapeutic intent were excluded.

*Context*: Any clinical, community, or home-based setting. No geographic restrictions were applied.

Additional inclusion criteria were: (1) original research reporting primary data; (2) assessment of at least one oral health outcome (clinical, microbiological, patient-reported, or biochemical); and (3) publication in a peer-reviewed journal. Exclusion criteria were: (1) narrative reviews, editorials, case reports, and expert opinions; (2) studies examining systemic effects of salt intake without oral health outcomes; and (3) studies where the salt-based intervention could not be isolated from other active components.

### 2.3. Study Selection

**Figure 2:**
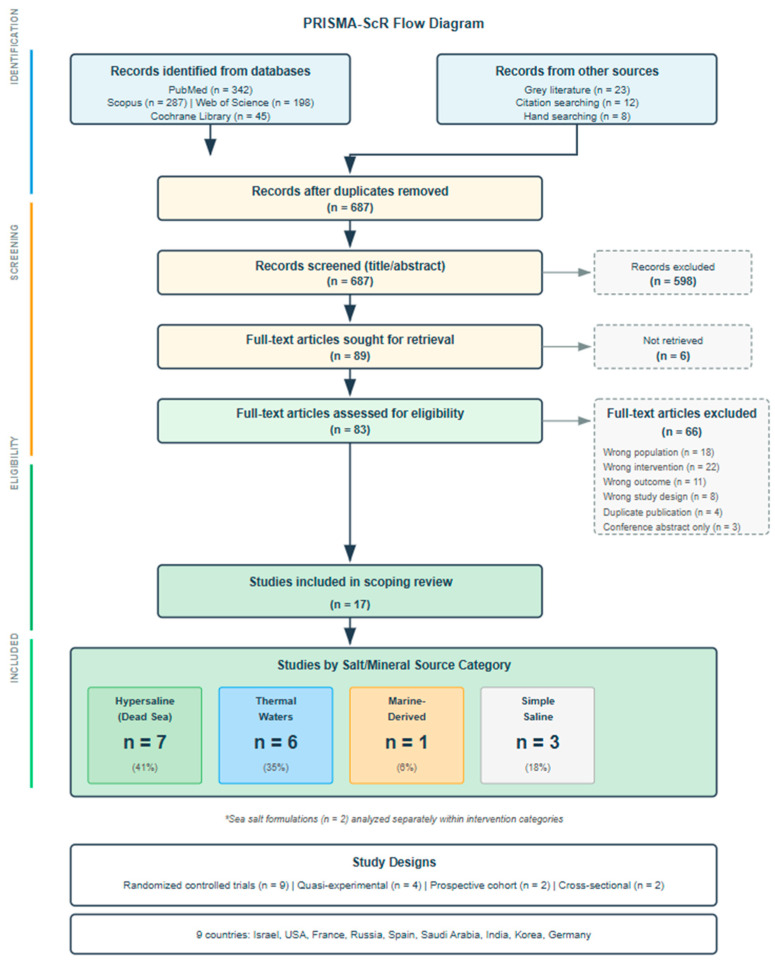
PRISMA-ScR flow diagram showing study selection process for salt-based oral health interventions. * Sea salt–based formulations (n = 2) were analyzed separately within the intervention categories and are therefore not included in the main salt/mineral source classification.

Study selection followed a two-stage process. In the first stage, two reviewers (EF, BS) independently screened titles and abstracts using Rayyan systematic review software (Available online: https://www.rayyan.ai. accessed on 26 November 2025). [18]. Studies deemed potentially relevant by either reviewer proceeded to full-text assessment. In the second stage, the same two reviewers independently evaluated full-text articles against pre-specified eligibility criteria. At both stages, reviewers met regularly to discuss uncertainties and resolve discrepancies through consensus. When agreement could not be reached, a third reviewer (GM) made the final determination. Reasons for exclusion at the full-text stage were documented systematically and are presented in the PRISMA flow diagram (Figure 2).

### 2.4. Data Extraction

Data extraction was performed independently by two reviewers (EF, BS) using a standardized extraction form developed a priori. The form was piloted on three randomly selected studies and refined to improve clarity and consistency. Extracted data included: bibliographic information, study design, setting, population characteristics, intervention details (water type, mineral composition when reported, concentration, application method, frequency, and duration), comparator, outcomes assessed, follow-up duration, and key findings. Discrepancies in extracted data were resolved through consensus discussion, with arbitration by a third reviewer (GM) when necessary. Given the descriptive nature of scoping reviews, study authors were not contacted for missing or unclear data. The full extraction template is available in the Supplementary Materials (Table S2).

### 2.5. Data Synthesis

Given the heterogeneity in study designs, populations, interventions, and outcomes, quantitative meta-analysis was not appropriate. Data were synthesized narratively following Synthesis Without Meta-analysis (SWiM) guidelines. Studies were grouped by intervention category (hypersaline/Dead Sea, thermal waters, marine-derived, simple saline, sea salt formulations) and outcome domain. Results are presented using descriptive statistics (frequencies, percentages, medians, ranges) and tabular summaries. The evidence map illustrates the distribution of studies across intervention types and outcome categories.

## 3. Results

### 3.1. Study Selection and Characteristics

The systematic search identified 915 records across four databases: PubMed (n = 342), Scopus (n = 287), Web of Science (n = 198), and Cochrane Library (n = 45). An additional 43 records were identified through grey literature searches (n = 23), citation searching (n = 12), and hand searching (n = 8). After removing 228 duplicates, 687 unique records underwent title and abstract screening, of which 598 were excluded as clearly irrelevant. Records were considered clearly irrelevant and excluded at the title and abstract screening stage primarily because they did not involve saline-based interventions applied to oral health conditions or addressed indications outside the scope of dentistry. Full-text assessment of 83 articles resulted in exclusion of 66 studies, leaving 17 studies that met all eligibility criteria (Figure 2). The included studies were published between 2007 and 2024 across nine countries. Sample sizes ranged from 10 to 93 participants (median 41), and follow-up periods ranged from 5 days to 3 months (median 4 weeks). Study designs included randomized controlled trials (n = 9), quasi-experimental studies (n = 4), prospective cohort studies (n = 2), and cross-sectional studies (n = 2). Studies were classified into five intervention categories: hypersaline Dead Sea derivatives (n = 7, 41%), thermal mineral waters (n = 5, 29%), simple saline solutions (n = 3, 18%), sea salt formulations (n = 2, 12%), and marine-derived solutions (n = 1, 6%). All thermal water studies except one investigated Castéra-Verduzan thermal water (France). Detailed study characteristics are presented in Table 1; distribution by intervention category is summarized in Table 2.

### 3.2. Outcomes and Comparators

**Figure 3:**
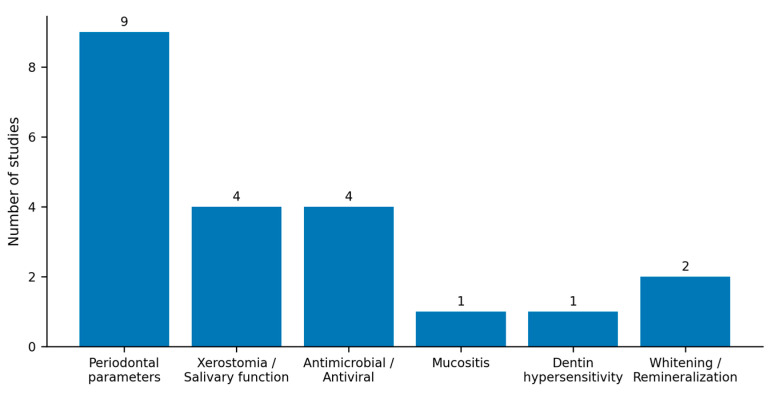
Distribution of outcome domains investigated across included studies.

Six outcome domains were identified: periodontal parameters (n = 9), xerostomia/salivary function (n = 4), antimicrobial/antiviral effects (n = 4), mucositis (n = 1), dentin hypersensitivity (n = 1), and whitening/remineralization (n = 2). Fourteen different assessment instruments were used, precluding quantitative synthesis. Chlorhexidine was the most common comparator (n = 6), followed by placebo/sham (n = 5), no treatment (n = 3), and other active comparators (n = 2). Three studies compared different formulations without external controls. Figure 3 summarizes the distribution of outcome domains investigated in the included studies.

This figure summarizes the distribution of outcome domains examined in the included studies, including periodontal parameters, xerostomia and salivary function, antimicrobial and antiviral effects, mucositis, dentin hypersensitivity, and whitening and remineralization outcomes. The visualization provides an overview of the evidence landscape without duplicating study-level results reported in the tables.

### 3.3. Findings by Intervention Category

#### 3.3.1. Hypersaline Dead Sea Derivatives

Seven studies reported mixed findings. Positive outcomes included significant viral load reductions (HSV-1, HCMV, EBV; *p* < 0.001) [20], bacterial toxin neutralization (leukotoxin −84%, endotoxin −40%) [21], periodontal improvements equivalent to chlorhexidine [22], and reduced radiation-induced mucositis severity [25]. However, Dead Sea products showed no advantage over conventional systems for tooth whitening [19] or enamel remineralization [24].

#### 3.3.2. Thermal Mineral Waters

Five studies showed inconsistent results for xerostomia: Alpöz et al. [27] found no difference versus placebo (with placebo performing better for some symptoms), while Skrinjar et al. [28] reported the highest quality-of-life effect size (0.52) for Buccotherm^®^. Toumassian et al. [29] demonstrated increased salivary mineralizing potential after 3 months. For gingivitis, Novozhilova et al. [30]—the only registered trial (NCT05623761)—reported significant gingival and bleeding improvements.

#### 3.3.3. Simple Saline Solutions

Three studies consistently reported equivalence to chlorhexidine for periodontal outcomes [32,33], with concentration-dependent antibacterial duration (5.8% saline matching chlorhexidine at 5 h) [33].

#### 3.3.4. Marine-Derived and Sea Salt Formulations

One marine-derived rinse study reported superior outcomes versus chlorhexidine [23]. Sea salt formulations showed mixed results: no significant benefit in one pilot study [34], but significant *S. mutans* reduction versus placebo in another [35]. Potential sources of bias across the included study designs are summarized in Table 3.

## 4. Discussion

This scoping review systematically mapped the available evidence on salt-based interventions for oral health. The findings reveal a nascent but heterogeneous evidence base characterized by geographic concentration, methodological limitations, and inconsistent results across similar interventions. The findings should be contextualized within the broader balneotherapy literature. Falagas et al. evaluated randomized controlled trials of balneotherapy across medical conditions, identified methodological limitations similar to those observed here: small sample sizes, inadequate blinding, and heterogeneous outcome measures [7].

Fioravanti et al. noted that while balneotherapy evidence is most robust for rheumatological and dermatological applications, other therapeutic domains remain understudied [36]. This pattern is confirmed by Cacciapuoti et al., whose review of thermal waters in chronic skin diseases identified substantially more controlled trials than the present review found for oral health applications—suggesting that oral health represents a particularly neglected domain within thermal medicine research despite shared mechanistic rationales [37]. To our knowledge, this represents the first scoping review to systematically map salt-based oral health interventions across the full compositional spectrum from geological mineral waters to artificial saline preparations.

Previous reviews have addressed isolated intervention types—such as thermal waters for dermatological conditions or balneotherapy for osteoarthritis without comprehensive mapping of salt-based approaches specifically for oral health outcomes [38,39]. The predominance of Dead Sea derivative research (41% of included studies) reflects established research infrastructure in Israel and commercial interest in hypersaline mineral products rather than inherently therapeutic ones.

Findings within this category were notably mixed: while Nowzari et al. reported significant antiviral effects and bacterial toxin neutralization [21,22], and Matceyevsky et al. demonstrated reduced radiation-induced mucositis severity [25], other studies found no advantage for tooth whitening or enamel remineralization [20,24]. This pattern suggests that Dead Sea derivatives may offer therapeutic potential for specific applications—particularly infection control and mucositis prophylaxis—while lacking broad-spectrum oral health benefits. The mechanistic basis for observed antiviral effects warrants further investigation, as reduction of oral herpesvirus loads could have implications for periodontal disease progression given established associations between viral reactivation and periodontitis [40]. Thermal water studies presented a particularly instructive pattern of contradictory findings.

Thermal water studies primarily addressed xerostomia, salivary function, and periodontal outcomes, with heterogeneous study designs and populations. For xerostomia management, Alpöz et al. found no significant difference between Buccotherm^®^ spray and placebo, with placebo actually performing better for mastication, swallowing, and speech symptoms [27]. In contrast, Skrinjar et al. reported the highest quality-of-life effect size (0.52) for the same product compared to alternative treatments [28]. These discrepant findings may reflect differences in study design (crossover versus parallel-group), outcome measures (VAS symptoms versus OHIP-14), or population characteristics (xerostomia etiology). Notably, Novozhilova et al.—the only registered trial among thermal water studies—demonstrated significant improvements in gingival indices and bleeding reduction, with enhanced dentin hypersensitivity outcomes when fluoride was added [30]. Toumassian et al. reported increased salivary mineralizing potential following three months of thermal water product use in post-COVID patients, though the absence of a placebo control limits causal interpretation. Critically, four of five thermal water studies investigated products from a single source (Castéra-Verduzan, France), limiting generalizability to the broader category of European thermal waters [29].

This concentration is notable given the extensive thermal medicine infrastructure in Germany, Italy, and other European countries with documented efficacy in dermatological applications [40], yet virtually no published research on oral health outcomes. Silva et al., investigating Portuguese Amarante thermal sulfur water, represent the sole exception and reported modest improvements in oral mucosal disease symptoms. The comparison with chlorhexidine efficacy provides important clinical context [26]. Lile et al., in their systematic review of chlorhexidine mouthrinses, documented consistent plaque and gingivitis reduction—establishing the benchmark against which alternative interventions must be evaluated [41]. Simple saline solutions demonstrated the most consistent findings across included studies relative to this standard. Collins et al. found saltwater rinse equivalent to chlorhexidine 0.12% following periodontal surgery, with no significant difference in gingival index reduction at 12 weeks [31]. Aravinth et al. reported comparable antimicrobial effects between salt water and chlorhexidine in schoolchildren [32]. Kamdem et al. additionally demonstrated concentration-dependent antibacterial duration, with 5.8% saline matching chlorhexidine efficacy at 5 h post-application [33].

These findings carry substantial implications for resource-limited settings where commercial antimicrobial formulations remain economically inaccessible. Moreover, growing concerns regarding chlorhexidine-induced microbiome disruption and potential cardiovascular implications through enterosalivary pathway interference suggest that simple saline solutions merit consideration as alternatives with more favorable safety profiles. However, the small sample sizes and short follow-up periods of included studies preclude definitive recommendations for clinical substitution. The marine-derived and sea salt formulation categories each contained too few studies for meaningful synthesis. The single marine-derived rinse study by Calvo-Guirado et al. reported superior periodontal outcomes versus chlorhexidine 0.2% and saline controls, but this finding requires replication before clinical interpretation [24]. Sea salt formulations showed mixed results: Hoover et al. found no significant differences in plaque or bleeding compared to standard oral hygiene [34], while Ballini et al. reported significant *S. mutans* reduction versus placebo [35]. However, these formulations combine salt with additional active ingredients (xylitol, lysozyme), precluding attribution of effects to the salt component specifically. Several methodological limitations characterized the included evidence base. First, 71% of studies enrolled fewer than 50 participants, limiting statistical power and precision of effect estimates. Second, only two studies exceeded four weeks follow-up, precluding assessment of sustained efficacy or long-term safety. Third, inadequate blinding was common—particularly problematic given the distinctive taste and appearance of many salt-based interventions. Fourth, outcome heterogeneity was substantial, with 14 different assessment instruments across 17 studies preventing quantitative synthesis. Finally, mineral composition was adequately reported in only four studies, limiting mechanistic interpretation and replication potential.

This scoping review has limitations inherent to its methodology. The restriction to peer-reviewed publications may have excluded relevant grey literature and introduced publication bias. The concentration of thermal water evidence from a single commercial source (Buccotherm^®^) may reflect publication bias toward industry-sponsored research.

Consistent with scoping review methodology and PRISMA-ScR guidelines, formal quality assessment was not performed, limiting interpretations of the reliability of findings [19]. The heterogeneity of interventions, populations, and outcomes precluded quantitative synthesis, and narrative approaches carry inherent subjectivity in evidence interpretation.

Future research priorities emerging from this evidence map include: (1) systematic investigation of European thermal waters beyond Castéra-Verduzan, utilizing standardized protocols and validated outcome measures; (2) adequately powered trials with extended follow-up periods (minimum 3–6 months) to assess sustained efficacy and safety; (3) head-to-head comparisons between intervention categories using common outcome measures to enable meta-analysis; (4) mechanistic studies characterizing the mineral composition requirements for therapeutic effects; and (5) investigation in underrepresented populations, particularly immunocompromised patients where preliminary mucositis findings suggest potential benefit [26]. Development of consensus core outcome sets for salt-based oral intervention trials would facilitate future evidence synthesis and clinical translation.

## 5. Conclusions

While preliminary findings suggest potential therapeutic applications—particularly for Dead Sea derivatives in periodontal care and mucositis prophylaxis—the limited number of studies, small sample sizes, and methodological heterogeneity preclude definitive clinical recommendations. Key research priorities include systematic investigation of European thermal waters using standardized protocols, larger adequately powered trials with extended follow-up, and development of consensus outcome measures. Until higher-quality evidence emerges, salt-based interventions should be considered experimental adjuncts requiring further validation before clinical implementation.

## Figures and Tables

**Table 1:** Characteristics of Included Studies.

Author	Water Type	Study Design	Population/Sample	Intervention	Control	Outcomes	Key Findings
HYPERSALINE WATER STUDIES (DEAD SEA REGION)							
Gurich et al. [19]	Hypersaline Dead Sea derivatives	Parallel-group double-blind RCT	50 adults (18–62 years)	Natural whitening regimen with Dead Sea salt-based products (strips, toothpaste, mouthwash)	Conventional peroxide-based whitening system	Objective tooth color assessment at 7, 10, and 14 days	No significant whitening with Dead Sea regimen; conventional peroxide-based treatment achieved measurable color improvement.
Nowzari et al., 2022a [20]	Hypersaline Dead Sea derivatives	Double-blind controlled trial	30 adults (25–35 years) with gingivitis and detectable oral viruses	Oral rinse containing Dead Sea salts used twice daily over 8-week period	Control rinse (distilled water)	Salivary detection of herpes viruses (HSV-1, HCMV, EBV)	Significant reduction of HSV-1, HCMV, and EBV viral loads vs. control (*p* < 0.001).
Nowzari et al., 2022b [21]	Hypersaline Dead Sea derivatives	Laboratory efficacy study	Mouse fibroblast cell cultures	Dead Sea salt solution exposure at therapeutic concentrations	Standard culture conditions	Cell viability and bacterial toxin neutralization	No cytotoxicity; significant reduction of leukotoxin (−84%), endotoxin (−40%), and glucan enzyme (−90%).
Rodriguez & Ajdaharian [22]	Hypersaline Dead Sea derivatives	Three-arm controlled trial	10 healthy volunteers	Commercial Dead Sea salt mouthwash (Oral Essentials brand)	Active control (chlorhexidine) and negative control (no rinse)	Standard periodontal indices (plaque, gingivitis, bleeding)	Dead Sea and chlorhexidine rinses equally reduced periodontal inflammation vs. no rinse; no significant difference between active treatments
Calvo-Guirado et al. [23]	Natural seawater (moderate salinity)	Crossover design RCT	93 dental students (19–42 years)	Marine-derived oral rinse (SEA 4 Encias brand)	Reference standard (0.2% chlorhexidine) and neutral control (saline)	Periodontal clinical parameters over 4-week trial	Marine rinse reduced plaque and gingival inflammation more than chlorhexidine and saline controls.
Ajdaharian et al. [24]	Hypersaline Dead Sea derivatives	Crossover enamel study	10 participants providing 300 tooth samples	Experimental sensitivity rinse with Dead Sea components and plant extracts	Commercial fluoride rinse (Sensodyne) and no-rinse control	Enamel surface microhardness recovery after demineralization	Dead Sea formulation showed no advantage; enamel remineralization was comparable across groups.
Matceyevsky et al. [25]	Hypersaline Dead Sea minerals	Prospective cohort study	54 cancer patients receiving head/neck radiotherapy	Prophylactic Dead Sea mineral products (oral rinse + topical cream)	Conventional supportive care	Radiation-induced oral and skin mucositis severity grading	Dead Sea mineral therapy reduced severe mucositis incidence and prevented treatment interruptions vs. standard care.
THERMAL WATER STUDIES							
Silva et al. [26]	Thermal sulfur water	Observational, longitudinal, comparative study	90 thermalists randomly allocated to groups	Thermal sulfuric natural mineral water of Amarante Thermal baths via gargles and oral showers for 14 days	Saline solution	Plaque index, gingival bleeding index, periodontal probing depth, oral mucosa disease symptoms	Greater pain reduction in TW_TA vs. saline (35.5% vs. 28.9%); OMD symptoms improved in both groups.
Alpöz et al. [27]	Thermal water (Castéra-Verduzan, France)	Single-blind crossover study	20 xerostomia patients (17 women, 3 men; age 43–75 years, mean 51.5)	Buccotherm^®^ spray 6 times daily for 14 days	Placebo (diluted tea solution with similar appearance)	Subjective xerostomia symptoms via VAS (10 items including dry mouth, difficulty swallowing, speech)	No significant difference in overall xerostomia relief vs. placebo; placebo showed lower VAS scores for mastication, swallowing, and speech (*p* ≤ 0.006).
Skrinjar et al. [28]	Thermal water (Castéra-Verduzan, France)	Open-label randomized controlled trial	60 drug-induced hyposalivation patients (45 women, 15 men; age 45–73 years, mean 64)	Buccotherm^®^ spray (n = 30) vs. Xeros^®^ mouthwash (n = 15) vs. marshmallow root (n = 15); 4 times daily for 2 weeks	Three-arm comparison	Quality of life (OHIP-14), dry mouth intensity (VAS)	Buccotherm^®^ had the greatest QoL effect size (0.52); VAS scores improved similarly across treatments (*p* < 0.05).
Toumassian et al. [29]	Thermal water (Castéra-Verduzan, France)	Prospective comparative study	80 post-COVID syndrome patients (dental students; mean age 21.5 years)	Group I (n = 46): Buccotherm^®^ toothpaste + mouthwash; Group II (n = 34): toothpaste + mouthwash + spray 3x/day; 3-month duration	Between-group comparison (no placebo)	Salivation rate, viscosity, pH, mineralizing potential, calcium and magnesium concentration	Mineralizing potential increased in both groups (Group I: 1.31→2.27; Group II: 1.28→2.87, *p* < 0.05); Group II showed greater improvements in salivation rate and mineral concentration.
Novozhilova et al. [30]	Thermal water (Castéra-Verduzan, France)	Double-blind parallel-group RCT	82 patients aged 20–25 years with gingivitis and dentin hypersensitivity	Toothpaste containing 46% Castéra-Verduzan thermal water (pH 8.8): Group TW (fluoride-free, n = 41) vs. Group TWF (with 1450 ppm NaF, n = 41); twice daily for 4 weeks	Between-group comparison	Modified Gingival Index, Bleeding Index, VAS and Schiff Scale for dentin hypersensitivity, Rustogi Modified Navy Plaque Index, salivary pH	Both groups showed improved gingival condition (MGI effect sizes: TW 0.99, TWF 1.71) and bleeding (BI: TW 3.17, TWF 2.64); dentin hypersensitivity decreased more in the TWF group (VAS effect size 3.28), while plaque index improved in both groups.
SIMPLE SALINE SOLUTION STUDIES							
Collins et al. [31]	Simple saline solution (artificial)	Randomized prospective double-blind study	37 chronic periodontitis patients	Saltwater mouth rinse following open flap debridement	0.12% chlorhexidine mouth rinse	Gingival Index, post-operative pain, mouth rinse satisfaction, matrix metalloproteinase activity	GI decreased from baseline to weeks 1 and 12 in both groups, with no significant between-group differences; saltwater was as effective as chlorhexidine.
Aravinth et al. [32]	Simple saline solution (artificial)	School-based randomized controlled trial	School children	Salt water rinse	Chlorhexidine mouth rinse	Dental plaque and oral microbial count	Saltwater rinse was effective as an adjunct to mechanical plaque control, with antimicrobial effects comparable to chlorhexidine
Kamdem et al. [33]	Simple saline solutions (artificial)	Cross-over clinical trial	10 participants (240 saliva samples)	Homemade saline solutions at different concentrations (2%, 5.8%, 23%)	0.1% chlorhexidine	Oral flora reduction and duration of effect	Dose-dependent antibacterial effect: 3 h (2%), 5 h (5.8%, comparable to chlorhexidine), and 7 h (23%, poorly tolerated).
SEA SALT FORMULATION STUDIES							
Hoover et al. [34]	Sea salt formulation	Pilot study	30 dental students aged 20–26 years	Sea salt, xylitol, and lysozyme mouth rinse for 30 days	Standard oral hygiene only	Turesky plaque index, gingival bleeding on probing	Overall plaque and gingivitis reduction did not differ between groups.
Ballini et al. [35]	Sea salt formulation	Randomized, double-blinded, placebo-controlled study	20 healthy adolescents	Combined mouth rinse with sea salt, xylitol, lysozyme, and menthol (H2Ocean)	Placebo rinse (mint-flavored water)	Plaque index, S. mutans levels	Significant reduction of S. mutans levels vs. placebo

Abbreviations: GI, gingival index; MGI, modified gingival index; QoL, quality of life; Quasi-exp, quasi-experimental; RCT, randomized controlled trial; VAS, visual analog scale. Notes: All thermal water studies used Castéra-Verduzan thermal water (France) as the mineral source.

**Table 2:** Distribution of included studies by intervention category.

Intervention Category	Studies (n)	Participants (Total n)	Percentage Countries	Countries
Hypersaline Dead Sea derivatives	7	154	41%	Israel, USA
Thermal/mineral waters	5	332	35%	France, Portugal, Turkey, Croatia, Russia
Marine-derived solutions	1	93	6%	Spain
Simple saline solutions	3	47 + NR	18%	USA, Cameroon, India

Participant totals reflect clinical studies only. One hypersaline study was laboratory-based and therefore excluded from participant counts. For one simple saline study, sample size was not reported in the original publication (NR).

**Table 3:** Overview of potential sources of bias across included study designs.

Study Design	Selection Bias	Performance/Observer Bias	Attrition Bias	Notes
Double-blind randomized controlled trials	Low	Low	Low-Moderate	Randomization and blinding reduce bias; small samples in some studies
Crossover randomized trials	Low-Moderate	Low-Moderate	Low	Potential carryover effects if washout is insufficient
Controlled clinical trials (non-randomized/three-arm)	Moderate	Moderate	Variable	Allocation not fully random; possible baseline differences
Open-label randomized trials	Low-Moderate	Moderate-High	Variable	Lack of blinding may influence subjective outcomes
Observational and cohort studies	Moderate-High	Moderate	Variable	Convenience sampling and confounding are possible
Laboratory efficacy studies	Not applicable	Not applicable	Not applicable	Non-clinical outcomes; limited direct clinical applicability

Note. This table provides a qualitative overview of potential sources of bias associated with the study designs included in this scoping review, including selection, performance/observer, and attrition-related biases. The overview is intended to support interpretation of the findings and does not represent a formal risk-of-bias assessment.

## Data Availability

No new data were created.

## Supplementary Materials

The following supporting information can be downloaded at: https://www.mdpi.com/article/10.3390/dj14010032/s1, Table S1: Complete Database Search Strategies; Table S2: Studies Excluded at Full-Text Screening with Reasons. Checklist S1: Preferred Reporting Items for Systematic reviews and Meta-Analyses extension for Scoping Reviews (PRISMA-ScR).

## Abbreviations

The following abbreviations are used in this manuscript:

BI	Bleeding Index
CMC	Carboxymethylcellulose
DH	Dentin Hypersensitivity
EBV	Epstein–Barr Virus
HCMV	Human Cytomegalovirus
HSV-1	Herpes Simplex Virus Type 1
JBI	Joanna Briggs Institute
MGI	Modified Gingival Index
NIH	National Institutes of Health
OHIP-14	Oral Health Impact Profile 14
OMD	Oral Mucosal Disease
OSF	Open Science Framework
PRISMA-ScR	Preferred Reporting Items for Systematic Reviews and Meta-Analyses extension for Scoping Reviews
QoL	Quality of Life
VAS	Visual Analog Scale
