# Supplementary material for: From Thermal Springs to Saline Solutions: A Scoping Review of Salt-Based Oral Healthcare Interventions
Source: Dent J (Basel). 2026 Jan 5;14(1):32. doi: 10.3390/dj14010032 (PMC12840170; doi:10.3390/dj14010032)
Supplement: Supplementary file 1 [file dentistry-14-00032-s001.zip › dentistry-4041253-supplementary.pdf]

Supplementary Materials

From Thermal Springs to Saline Solutions: A Scoping Review of Salt-Based Oral Healthcare Interventions

Table S1. Complete Database Search Strategies

Searches conducted December 2024, updated November 2025. No date restrictions applied.

PubMed/MEDLINE

(("thermal water*"[Title/Abstract] OR "mineral water*"[Title/Abstract] OR "saline solution*"[Title/Abstract] OR "salt water"[Title/Abstract] OR "Dead Sea"[Title/Abstract] OR "seawater"[Title/Abstract] OR "marine water*"[Title/Abstract] OR "brackish water*"[Title/Abstract] OR "hypersaline"[Title/Abstract] OR "thermal spring*"[Title/Abstract] OR "hot spring*"[Title/Abstract] OR "mineral spring*"[Title/Abstract] OR "spa therapy"[Title/Abstract] OR "balneotherapy"[Title/Abstract] OR "crenotherapy"[Title/Abstract] OR "halotherapy"[Title/Abstract] OR "brine"[Title/Abstract])

AND

("oral health"[MeSH Terms] OR "oral"[Title/Abstract] OR "dental"[Title/Abstract] OR "periodontal*"[Title/Abstract] OR "gingiv*"[Title/Abstract] OR "periodont*"[Title/Abstract] OR "oral mucosa*"[Title/Abstract] OR "mouth"[Title/Abstract] OR "stomatitis"[Title/Abstract] OR "mucositis"[Title/Abstract] OR "dental plaque"[Title/Abstract] OR "oral bacteria*"[Title/Abstract] OR "halitosis"[Title/Abstract] OR "dental caries"[Title/Abstract] OR "mouthwash*"[Title/Abstract] OR "mouth rins*"[Title/Abstract] OR "oral hygien*"[Title/Abstract]))

Filters: Clinical Trial, Randomized Controlled Trial, Controlled Clinical Trial, Comparative Study; Languages: English, Italian, Spanish, French, German, Portuguese

Scopus

TITLE-ABS-KEY("thermal water*" OR "mineral water*" OR "saline solution*" OR "salt water*" OR "seawater" OR "sea water" OR "marine water*" OR "brackish water*" OR "hypersaline" OR "Dead Sea" OR "thermal spring*" OR "hot spring*" OR "mineral spring*" OR "spa therapy" OR "balneotherapy" OR "crenotherapy" OR "halotherapy" OR "brine")

AND

TITLE-ABS-KEY("oral health" OR "dental" OR "dentistry" OR "periodontal*" OR "gingiv*" OR "periodont*" OR "oral mucosa*" OR "mouth" OR "oral cavit*" OR "stomatitis" OR "mucositis" OR "oral surg*" OR "dental plaque" OR "oral bacteria*" OR "halitosis" OR "oral infection*" OR "dental caries" OR "tooth" OR "teeth" OR "gum*" OR "mouthwash*" OR "mouth rins*" OR "oral hygien*")

Limits: DOCTYPE (ar, re); LANGUAGE (English, Italian, Spanish, French, German, Portuguese)

Web of Science Core Collection

TS=("thermal water*" OR "mineral water*" OR "saline solution*" OR "salt water*" OR "seawater" OR "sea water" OR "marine water*" OR "brackish water*" OR "hypersaline" OR "Dead Sea" OR "thermal spring*" OR "hot spring*" OR "mineral spring*" OR "spa therapy" OR "balneotherapy" OR "crenotherapy" OR "halotherapy" OR "brine")

AND

TS=("oral health" OR "dental" OR "dentistry" OR "periodontal*" OR "gingiv*" OR "periodont*" OR "oral mucosa*" OR "mouth" OR "oral cavit*" OR "stomatitis" OR "mucositis" OR "oral surg*" OR

"dental plaque" OR "oral bacteria*" OR "halitosis" OR "oral infection*" OR "dental caries" OR "tooth" OR "teeth" OR "gum*" OR "mouthwash*" OR "mouth rins*" OR "oral hygien*")

Refined by: DOCUMENT TYPES (Article OR Clinical Trial); LANGUAGES (English OR Italian OR Spanish OR French OR German OR Portuguese)

Cochrane Central Register of Controlled Trials (CENTRAL)

#1 ("thermal water*" OR "mineral water*" OR "saline solution*" OR "salt water*" OR "seawater" OR "sea water" OR "marine water*" OR "brackish water*" OR "hypersaline" OR "Dead Sea" OR "thermal spring*" OR "hot spring*" OR "mineral spring*" OR "spa therapy" OR "balneotherapy" OR "crenotherapy" OR "halotherapy" OR "brine"):ti,ab,kw

#2 ("oral health" OR "dental" OR "dentistry" OR "periodontal*" OR "gingiv*" OR "periodont*" OR "oral mucosa*" OR "mouth" OR "oral cavit*" OR "stomatitis" OR "mucositis" OR "oral surg*" OR "dental plaque" OR "oral bacteria*" OR "halitosis" OR "oral infection*" OR "dental caries" OR "tooth" OR "teeth" OR "gum*" OR "mouthwash*" OR "mouth rins*" OR "oral hygien*"):ti,ab,kw

#3 #1 AND #2

Additional Sources

Grey literature searches were conducted in ClinicalTrials.gov, WHO International Clinical Trials Registry Platform (ICTRP), and OpenGrey. Forward and backward citation tracking was performed for all included studies. Expert consultation with balneotherapy researchers was conducted to identify unpublished or in-press studies.

Table S2. Studies Excluded at Full-Text Screening with Reasons

Following title and abstract screening, full-text articles were assessed for eligibility. Studies were excluded for the following reasons:

Reason for Exclusion	Description	Number of Studies
Thermal effects focus	Studies examining heat/temperature effects rather than therapeutic mineral water applications	n = 4
Oral irrigation without saline focus	Studies on oral irrigation devices using plain water without specific saline or mineral content	n = 3
Oral temperature measurement	Studies measuring oral temperature rather than evaluating water-based therapeutic interventions	n = 2
Insufficient sample size	Case reports or studies with fewer than 5 participants	n = 1
**TOTAL EXCLUDED**		**n = 10**

Note: All exclusion decisions were made independently by two reviewers (EF, BS) with disagreements resolved through discussion or consultation with a third reviewer (GM). Inter-rater agreement was substantial (Cohen's $\kappa = 0.82$).
